# Surgical Management of Cystic Pelvic Hydatid Bone Disease Using Additively Manufactured Customized Implants for Salvage Reconstruction: A Report of Two Cases

**DOI:** 10.7759/cureus.63559

**Published:** 2024-07-01

**Authors:** Rodica Marinescu, Carmen Michaela Cretu, Stefan Ciumeica, Laptoiu Dan Constantin

**Affiliations:** 1 Orthopedic Surgery, Carol Davila University of Medicine and Pharmacy, Bucharest, ROU; 2 Clinical Parasitology, Colentina Clinical Hospital, Bucharest, ROU; 3 Orthopedics, Colentina Clinical Hospital, Bucharest, ROU

**Keywords:** additive manufacturing, customized implant, surgical and non-surgical management, severe bone destruction, cystic echinococcosis

## Abstract

The diagnosis and treatment of pelvic bone hydatidosis (BH) present substantial challenges for orthopedic surgeons, requiring collaboration with parasitologists, radiologists, pathologists, and engineers. Surgical treatment selection depends on factors such as the extent of bone loss, soft tissue management, previously applied therapies, and local colonization status.

This report details the advanced management of two young patients diagnosed late with severe cystic pelvic BH, an atypical presentation due to their geographic origin and age. Following extensive diagnostic assessments, including serology and 3D imaging, the patients underwent a two-step surgical intervention. The initial surgery involved extensive debridement and the placement of a poly-methyl-methacrylate spacer, followed by a second procedure utilizing a custom-made, tri-flanged implant for definitive pelvic reconstruction. The custom implant, designed via an electron beam melting process, successfully restored hip functionality and anatomy, as evidenced by improvements in functional scores and post-operative imaging. Short-term monitoring confirmed the integration of the implant and the absence of infection recurrence, demonstrating the approach's effectiveness.

These cases highlight the potential of using additive manufacturing (AM) to create patient-specific implants for managing complex hip cases and emphasize the necessity for early detection and a multidisciplinary approach in treatment planning.

## Introduction

Bone osseous cystic echinococcosis, often known as bone osseous hydatidosis (BH), is one of the most complex and late-diagnosed forms of echinococcosis [1]. It is caused by the larval form of *Echinococcus granulosus*, which is particularly prevalent in geographically defined endemic areas [2]. Although Romania is not typically considered an endemic area in Europe, there is a documented incidence of 4%-5% cases per 100,000 inhabitants, especially in the north-east and southern regions [3]. In endemic regions, cystic echinococcosis (CE) poses a significant burden and remains a public health concern, with over 20% of reported cases occurring in patients younger than 19 years [4]. BH is typically diagnosed between the fifth and sixth decades of life, while the cases discussed here involve patients in their third and fourth decades, indicating early exposure. The most common location for BH is the vertebrae, accounting for approximately 50% of cases, followed by the pelvis and long bones of the lower limb. Previous reports have considered the pelvis as the second most common location for BH, with an occurrence rate of approximately 28% [5]. The ilium is the preferred site for pelvic hydatidosis, as previously noted by Zlitni et al. The same study reported an infection rate of 30%, which is a crucial factor in the treatment protocol and patient prognosis [6].

Bone hydatid disease primarily affects trabecular bone and can progress to the destruction of cortices, leading to the expansion of the disease into adjacent soft tissues. The slow progression rate often results in long periods of silent development, leading to cases that go undetected clinically. As a result, patients are usually diagnosed at an advanced stage when considerable bone destruction already exists, and the resulting disability is severe. The disease has a high recurrence rate, necessitating complex anti-helminthic chemotherapy, surgical treatment, and long-term monitoring. Serological tests, including ELISA, are used to confirm the disease. WB confirmatory tests should be used when ELISA test results are negative. Viability tests for protoscoleces and PCR genotyping can be added to surgical samples [7]. Diagnosing and treating pelvic hydatidosis present a significant challenge for orthopedic surgeons, who must collaborate within a multidisciplinary team that includes parasitologists, radiologists, and pathologists. Medical engineers also play a decisive role, especially in cases where customized implants are needed to address bone defects and restore hip function effectively. Advanced cases require significant surgical intervention, often necessitating the use of innovative techniques such as 3D-printed custom implants. The integration of bioengineering for the 3D digital reconstruction of patient bone structures and the use of additive manufacturing (AM) metal-based processes have revolutionized the customization of implants that adapt precisely to the complex topology of the affected bone [8,9]. Despite the high costs associated with these advanced technologies, their ability to enhance osseointegration and address intricate anatomical features justifies their use, particularly in challenging cases of pelvic echinococcosis [10,11]. Currently, the primary management of advanced BH involves both surgical intervention and antiparasitic chemotherapy, with a notable study by Luan et al. documenting a series of patients treated with 3D-printed implants, showing a recurrence rate of about 30% [12].

Our study aims to provide a comprehensive review of managing severe pelvic bone destruction caused by cystic echinococcosis, especially in late-diagnosed scenarios, highlighting the critical role of AM in the surgical strategy and offering insights into future treatment pathways for this debilitating condition [13].

## Case presentation

The proposed treatment method involves confirming pelvic hydatid bone disease through serological and radiological examinations, including computed tomography (CT) with 3D reconstruction. The defects are then evaluated and classified, and custom metal implants, hemipelvis implants, and polymer replicas and implants are manufactured for surgical rehearsal, surgery, and long-term monitoring. Surgery is performed, followed by long-term monitoring of the patients. This approach combines standard disease management with the design and additive manufacturing of tailored implants based on CT data and 3D reconstructions, and specific surgical steps for implant positioning and fixation to restore bone loss and reconstruct pelvic anatomy and function. Two complex cases with late diagnoses in young patients are presented to emphasize the significance of this integrated approach.

Case 1

**Figure 1:**
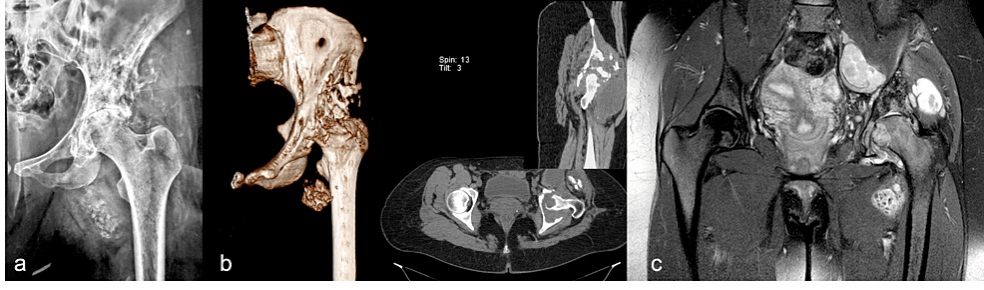
Clinical Case 1: preoperative imaging Preoperative X-ray (a); CT scan showing bone destruction of acetabulum-anterior and posterior walls and iliac (Enneking bone zone Ic+II) of a late-diagnosed patient (b); and preoperative MRI aspect with confluent pseudo-tumoral masses (c).

A 28-year-old female patient with a history of multiple liver hydatidosis diagnosed at the age of four underwent anti-helminthic therapy until the age of 12 years. In 2017, the patient had comprehensive serological tests for hydatidosis, which all yielded negative results. In 2018, the patient experienced a left knee injury from falling down stairs, leading to ligament surgery with a difficult recovery and significantly reduced knee mobility. Additionally, the patient experienced hip pain and disability. The orthopedist initially overlooked this issue, but in 2021, a hip X-ray identified the problem as hip osteoarthritis. Further investigations, including MRI and biopsy, revealed hydatid echinococcosis. The patient was treated with systemic albendazole chemotherapy and ultrasound-guided aspiration/inactivation using hypertonic saline solution to address the anteromedial hip thigh collection. Despite these interventions, the hip pain and disability gradually worsened. The patient had a pain-free mass with a liquid consistency extending from the iliac area and running along the medial thigh aspect, and bacterial contamination by *Staphylococcus capitis* was identified. Radiographic investigations revealed multiple osteolytic lesions dispersed in the acetabulum, femoral head, and ilium regions, with narrowing of the hip joint space and irregular cystic lesions in the soft tissue with calcifications (Figure 1).

Ilium destruction, with lateral cortices eroded by approximately 5 cm, and a lytic confluent area extending toward the sacroiliac joint were observed on MRI examination. A CT scan with 3D reconstruction showed severe bone destruction affecting the acetabulum-anterior and posterior walls and iliac (Enneking-like bone zone Ic+II destruction), potentially unfit for reconstruction with a conventional endoprosthesis (Figure 1, Panels b and c).

Case 2

**Figure 2:**
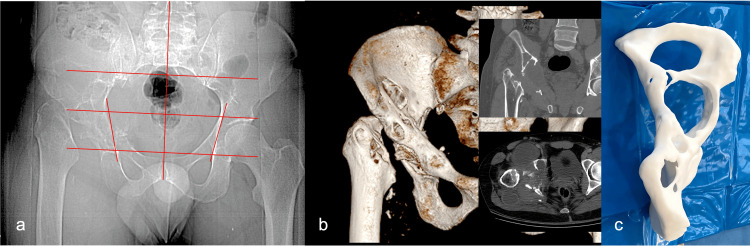
Clinical Case 2: preoperative imaging studies Initial X-ray evaluation (a); CT scan showing multiple collections and cysts (b); and hemipelvis PLA (polylactic acid) 3D-printed replica with evidence of severe bone loss (c).

A 38-year-old male from a southern endemic area experienced right pelvic swelling, mild pain, and progressive hip functional deficit in 2014. Three years later, a plain X-ray examination suggested bone tuberculosis, and the patient underwent extensive debridement, femoral head resection, and specific antituberculosis therapy. However, bone destruction continued to advance toward the gluteal region, and the patient was referred to our hospital parasitology clinic in 2019. ELISA serological investigations eventually diagnosed hydatidosis, and specific albendazole therapy was initiated. At admission, the patient complained of increased hip pain, restricted range of motion, severe limping with a 5 cm length discrepancy, and discharge of seropurulent secretion from a gluteal catheter. Pelvic area X-ray examination showed the absence of the femoral head and neck and several lytic irregular lesions in the acetabular area, extending proximally toward the ilium and distally toward the ilio-pubic and ilio-ischial-pubic rami (Figure 2, Panel a). A CT scan revealed an acetabular defect and a large lytic area extending toward the ilium and smaller lytic areas into the ilio-ischial branch (Figure 2, Panels b and c). The extensive destruction of the acetabulum and iliac bone zone Enneking Ic+II, along with the poor quality of the remaining bone, led to the decision to use a customized implant.

Surgical technique protocol

**Figure 3:**
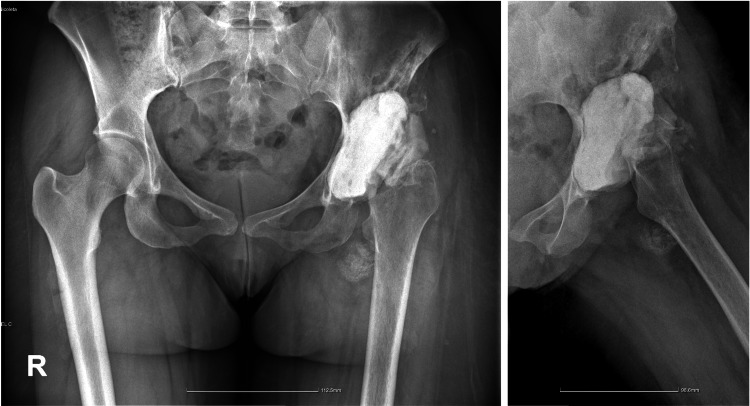
Postoperative X-ray: first surgical step This is an example of the postoperative X-ray aspect after the surgical step of debridement with the polymethyl-methacrylate mixed with an antibiotic-spacer in place (Case 1).

The first step of the surgical protocol involved extensive debridement and inactivation using a hypertonic chloride solution (approx. 5 liters). Necrotic bone and devitalized soft tissues were removed in an oncologic-like manner, leaving a large anfractuous pelvic defect after head and femoral neck resections. A bone cement spacer loaded with antibiotics (2 g of vancomycin in 40 mg acrylic cement), custom-made intra-operatively, was left in place at the end of the procedure (Figure 3).

**Figure 4:**
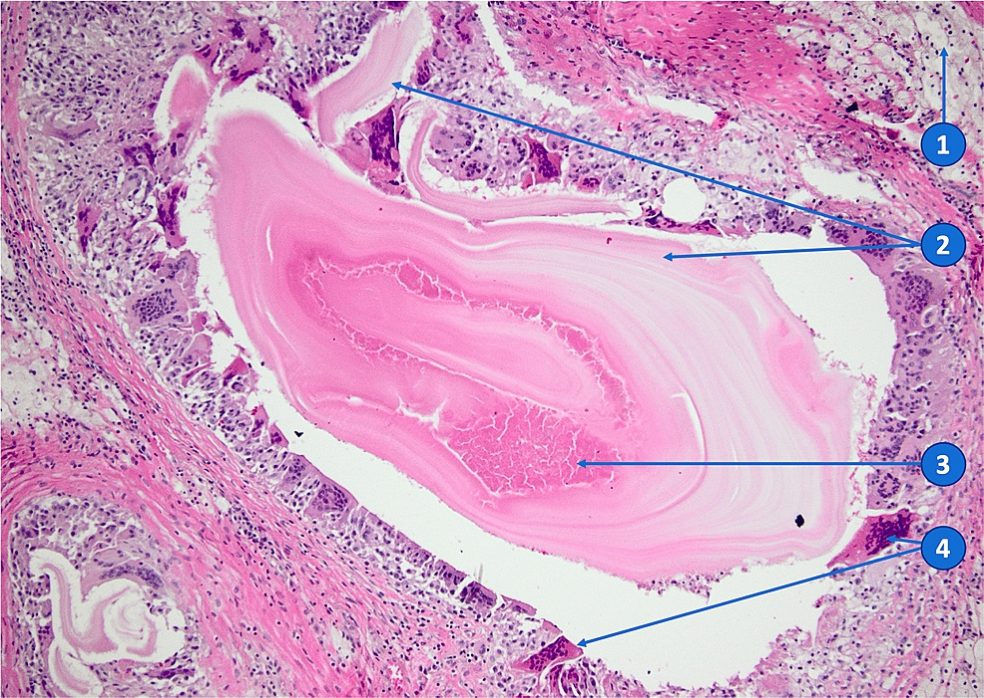
Anatomopathologic findings: foreign body granulomatous inflammation associated with proliger membranes (HE x100) (Case 1) (1) Foamy macrophages, (2) proliger membranes, (3) necrotic detritus (with degraded protoscoleces), and (4) multinucleated foreign body giant cells.

An antiseptic lavage was performed to prevent bacterial contamination. The histological specimen (Figure 4) was positive for BH.

**Figure 5:**
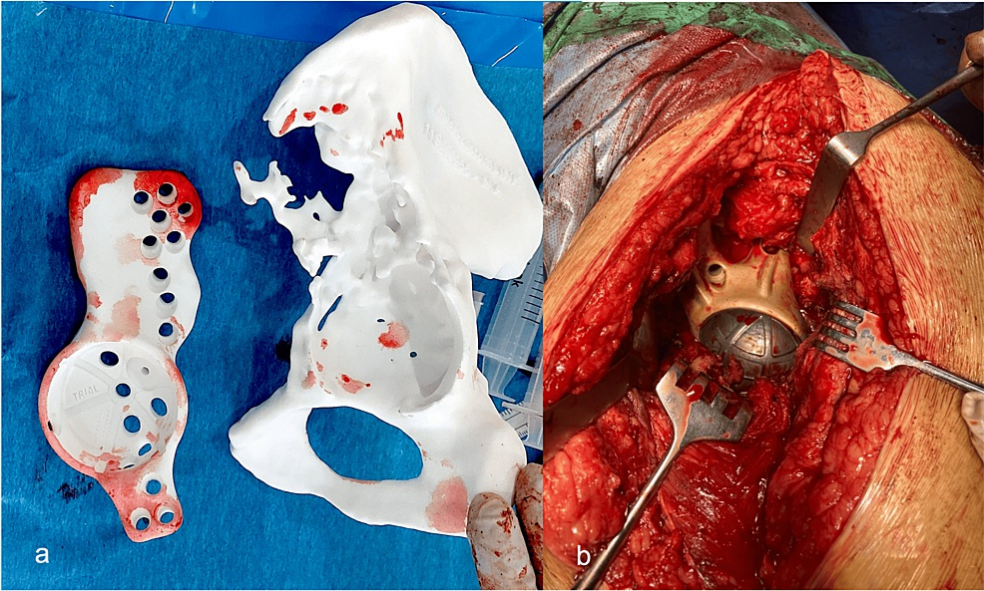
Intraoperative images: second surgical step (Case 1) Intraoperative use of the sterilized 3D-printed model (a) for implant adaptation after bone debridement and grafting during reconstruction, which is the second surgical step (b). The model also guides several intraoperative stages.

In all of our patients with sepsis, recommended systemic intravenous antibiotic therapy was followed by oral therapy. The patients were prescribed a combination of albendazole and praziquantel for six weeks before the next surgery, according to the parasitologist's recommendations. In the second surgical step, a customized prosthesis was implanted using sterilized 3D-printed replicas of the hemipelvis and implant to ensure proper accommodation of the implant within the bone bed (Figure 5, Panels a and b). If necessary, additional debridement and gentle reaming of the acetabulum were performed. Before implant positioning, morselized allograft chips were impacted into the remaining ilium and acetabular defects.

**Figure 6:**
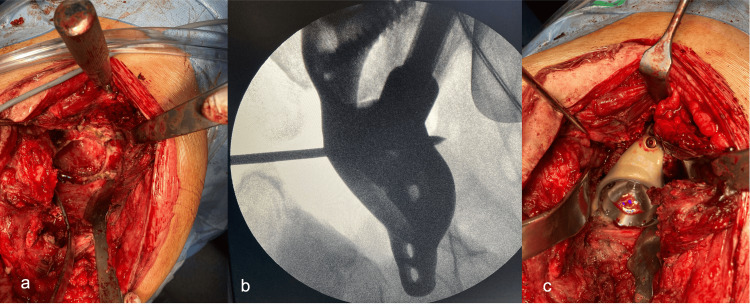
Intraoperative images: second surgical step (Case 2) Intraoperative aspects with debridement (a), fluoroscopic guidance (b), and implantation of a customized implant (c).

Figure 6 presents the main fixation of an AM implant, achieved with the iliac stem along the linea arcuata under fluoroscopic guidance, as well as additional fixation with the iliac and ischial flanges. Antibiotic bone-loaded substitutes (Stimulan®, Biocomposites) were added to the surrounding area [14].

The follow-up plan involved visits every three to six months for the first two years and then annually thereafter. Documentation included functional scores, potential complications, and radiographic assessments. To ensure impartiality, the Musculoskeletal Tumor Society (MSTS) score, which is used for patients with custom-made implants due to similarities with oncological cases in the follow-up protocol, along with the Harris hip score (HSS) [15], was used for functional evaluations.

**Figure 7:**
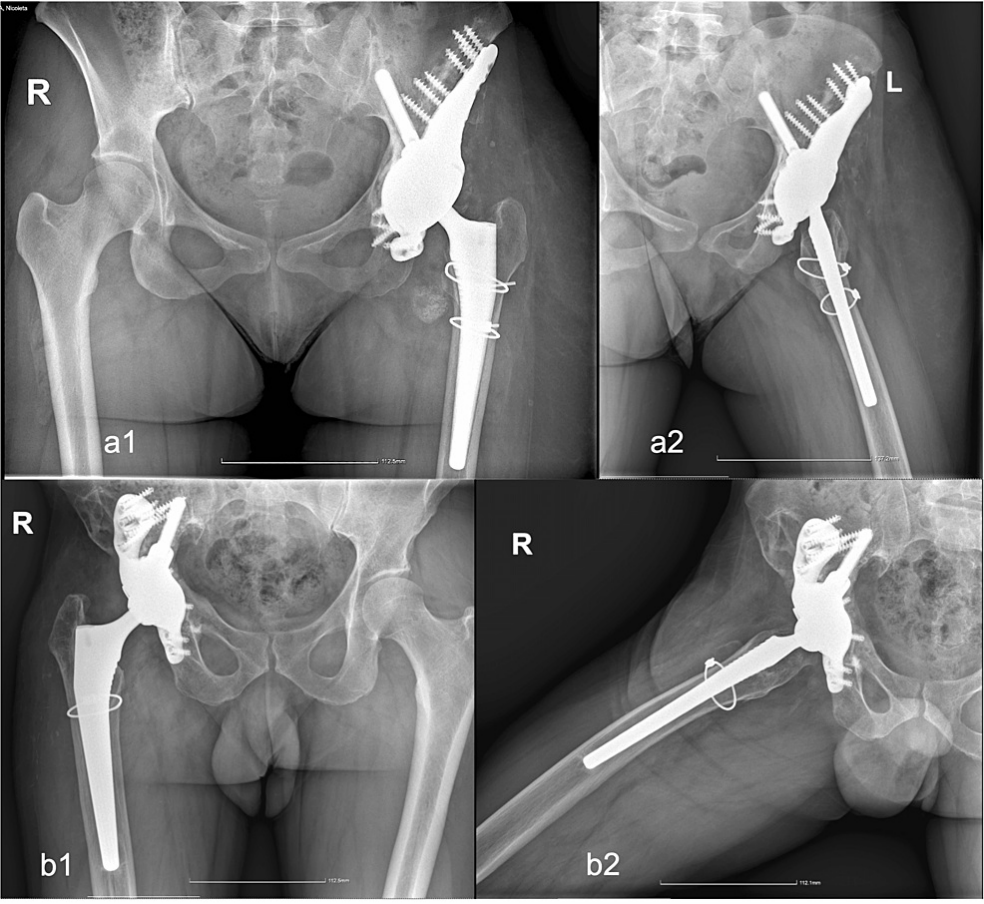
Radiographic control at follow-up Radiographic investigation at one-year follow-up (a: Case 1) and two years (b: Case 2).

After surgery, patients initiated non-weight-bearing walking for two months, followed by a gradual transition to weight-bearing over the subsequent two months. By three months post-operative, they had achieved full weight-bearing walking. Postoperative radiography confirmed restoration of the hip rotation center and correct implant positioning. By six months, the patients could walk without canes and reported no pain, with an improved range of motion (Figure 7).

## Discussion

Table 1 summarizes the clinical aspects of the specific cases presented, validating the approach based on customized AM implants. Each case, analyzed in this study, had distinct features. In the first patient, BH developed after a 10-year break from anti-helminthic treatment for liver hydatid cysts. It is challenging to determine if liver and bone colonization happened concurrently during childhood or if the bone location was secondary due to the potential injury of a liver cyst during labor, as the patient missed follow-up for almost a decade. The absence of visceral echinococcosis in the second case confirmed the primary bone hydatidosis diagnosis.

**Table 1:** Clinical results of patients Pre-op and post-op: Pre-operative and post-operative functional scores; MSTS: Musculoskeletal Tumor Society score; HSS: Harris hip score.

Case post-op	Age (years)	Gender	Extra pelvic infection	Enneking pelvic classification	Treatment protocol	Follow-up (years)	MSTS pre-op	MSTS post-op	HSS pre-op	HSS post-op
1	28	F	Liver	Grade Ic+II	Two-stage customized hip implant	1	9	23	10	67
2	38	M	No	Grade Ic+II	Two-stage customized hip implant	2	9	26	10	65

Patients with pelvic hydatid disease often experience pain, claudication, sinus formation, and compression-related symptoms [16]. Initially, pain may not be severe or specific, leading to a progressive functional deficit being the patient's primary complaint. As bone destruction advances, these functional deficits gradually worsen, leading to varying degrees of disability. Late-diagnosed cases of bone hydatid disease typically present with limping, difficulty walking, and severely diminished range of motion.

Radiological examination is the starting point for the BH diagnosis. The radiological features of osseous hydatidosis are not specific and are prone to confusion with bone tuberculosis, bone metastases, or giant cell tumors [17]. Confusion can occur in the imaging diagnosis of bone hydatidosis, leading to an alarmingly low preoperative diagnosis rate of approximately half of the cases. This might lead to an incorrect case approach, potentially increasing the recurrence disease rate. An elevated eosinophil count should alert orthopedic surgeons to the possibility of BH and prompt further investigation, particularly in patients from endemic areas.

Usually, plain X-rays document multiple, various grades of bone destruction, with lytic-type confluent, irregular lesions, marginal osteosclerosis, and no periosteal reaction. Bone loss limits are not well-defined, and some bone calcifications may be observed. Irregular low-density shadows may occur when soft tissues have invaded. Soft tissue calcification, when associated with previous radiological signs, is considered highly suggestive of BH [18]. The articular space of the affected sacroiliac or hip joint may become progressively narrow and even disappear. The radiological presentation of pelvic hydatidosis is influenced by factors such as cyst location, age, and associated complications such as secondary infection and cyst rupture. Therefore, a definitive diagnosis requires a combination of clinical history and additional imaging.

The CT exam revealed branch-like bone cysts that replaced the bone gradually and involved surrounding tissues. 3D CT scans are crucial for preoperative planning and implant selection in cases of severe bone destruction. MRI is highly valuable in diagnosing bone hydatidosis, with the ability to differentiate between parent and daughter cysts and accurately depict the expansion of cysts into soft tissue [19].

The recommended treatment for BH involves systemic anthelmintic chemotherapy, local surgery, and radiotherapy for inoperable patients. Parasitology physicians decide on the anti-helminthic treatment for pelvic hydatidosis. Albendazole is the most frequently used drug, administered at a dosage of 10 mg/kg/day with fatty meals and silymarin to improve bioavailability. It is recommended for cases involving the pelvic bone, iliac bone, and hip joint. Treatment typically begins at least 21 days pre-operatively and continues for a minimum of six months post-operatively, sometimes for years.

Some researchers recommend long-term albendazole therapy for pelvic hydatidosis with extensive destruction, where complete resection is uncertain, to decrease recurrence rates. Patients capable of tolerating the medication before and after surgery may benefit from this approach as it may halt the progression of the parasite and improve survival rates [16]. Our patients were chosen for this method due to the severity and uncertainty of their disease. However, during surgery, appropriate resection is challenging due to the difficulty in defining the affected bone landmarks, which are often near high-risk structures. Additionally, estimating the age of the disease is difficult due to late diagnosis and severe local destruction.

Due to the high recurrence rate of the disease, surgical management should emulate oncological approaches, categorizing surgical margins as wide, marginal, or intralesional. However, accurately defining the boundaries of the disease is challenging because of its unique development in bone, which lacks a distinct pericystic membrane [16]. In our experience, the affected bone appears fragile, white, and crumbly, making it easily removable using a regular curette.

For optimal and long-lasting outcomes, wide resection of the bone with all surrounding affected tissues is recommended as incomplete resection increases the risk of recurrence [20]. Regardless of the surgical technique used, additional precautions are required. These include carefully protecting the surrounding soft tissue with gauze soaked in a 20% sodium chloride solution and meticulously cleaning the peripheral remnants of the bone defect with a speed burr whenever feasible.

Adding bone-filling cement may further decrease the recurrence rate as the temperature elevation during polymerization is high and proven to be effective for daughter cysts [19]. Similar therapy, cement with antibiotic spacers, is mandatory in cases of bacterial colonization as part of a two-step surgery protocol.

For extensive and irregular bone defects, a customized implant may be the optimal solution for restoring the patient's hip joint. This technique has several advantages. First, it allows the surgeon to effectively restore the hip joint function and kinematics. Second, it enables the complete resection of all affected bones without concerns about uncontained defects. Third, the primary implant stabilization was enhanced through supplementary fixation and precise positioning of the device. Encouraging results have been reported regarding the restoration of hip functionality and implant survival [20]. However, it is important to note that the costs associated with these implants are considerable, and there is a necessary lead time for design and production based on a specific patient's bone defect and accurate mapping of areas with good bone quality for proper fixation.

The use of additional implant coatings, such as silver nanoparticles, is recommended in cases with documented infections. Given the heightened risk of invasive procedures in hydatidosis cases before implantation, silver-nanocoating technology may help prevent periprosthetic joint infections. Moreover, in cases with confirmed infections and those involving extensive bone defects, where there is an increased risk of incomplete implant bone coverage, an additional silver nanoparticle coating may be beneficial. This coating inhibits bacterial growth, exhibits potent antibiofilm properties, and does not compromise implant fixation or osseointegration.

Allografts are also essential for managing bone defects in BH cases, with both morselized and structural allografts reporting favorable outcomes in previous studies [2]. In the cases discussed here, morselized impacted allografts were used to improve implant fixation and replenish bone stock. Radiological assessments conducted one and two years post-operatively indicated successful graft incorporation and restoration of the bone bed.

## Conclusions

This case report presents a comprehensive approach to improve patient outcomes and quality of life for individuals with cystic pelvic hydatid disease, whether primary or secondary. The study focuses on young patients who experienced delayed diagnoses and significant bone loss, which cannot be addressed with standard implants.

Surgeons should be aware of prolonged nonspecific symptoms and suggestive imaging findings, particularly in patients from endemic regions, and conduct a thorough investigation. The recommended treatment includes anthelminthic chemotherapy and surgical intervention, with customized implants required for severe bone loss cases. Preoperative antiparasitic chemotherapy and additional implant coatings, such as silver nanoparticles, are essential for preventing recurrence and periprosthetic joint infections. Allograft incorporation is crucial for successful bone-stock restoration. Long-term monitoring is necessary to assess the effectiveness of the treatment and address potential complications.
